# Minimizing sampling error using a unique ink-stained reflectance confocal microscopy-guided biopsy technique to diagnose a large lentigo maligna

**DOI:** 10.1016/j.jdcr.2022.03.031

**Published:** 2022-03-31

**Authors:** Joanna Ludzik, Claudia Lee, Alexander Witkowski, Jonathon Hetts

**Affiliations:** a Department of Telemedicine and Bioinformatics, Jagiellonian University Medical College, Krakow, Poland; b Department of Dermatology, Oregon Health and Sciences University, Portland, Oregon; c School of Medicine, University of California Riverside, Riverside, California

**Keywords:** confocal microscopy, lentigo maligna, RCM, RCM-guided biopsy, reflectance confocal microscopy, RCM, reflectance confocal microscopy

## Clinical presentation

**Fig 1:**
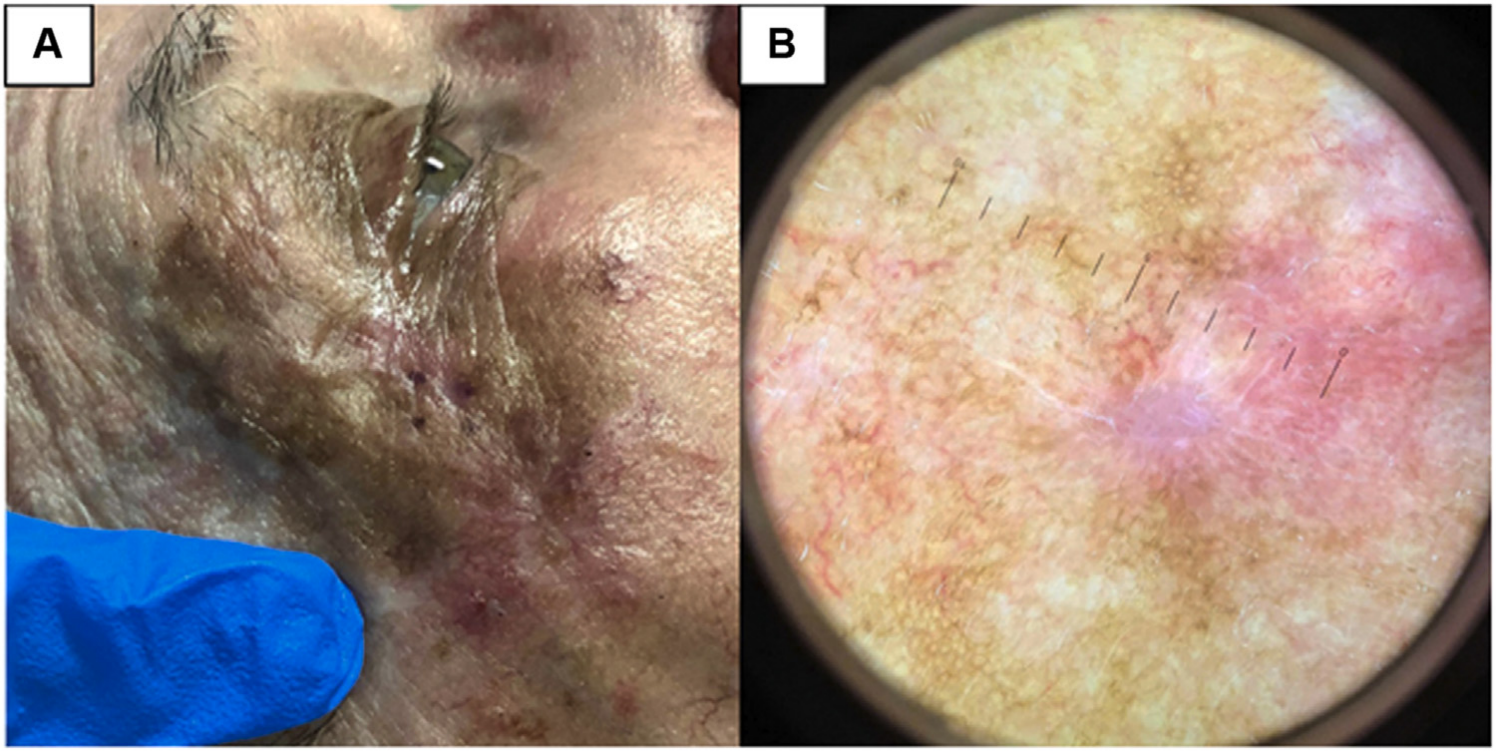
**A**, Clinical image of 5.5-cm × 5.0-cm brown patch with variegated pigmentation involving the right zygomatic area with presence of scar postbiopsy. **B,** Dermatoscopic image of the scar, showing mild perifollicular hyperpigmentation near the lateral border of the scar.

An 88-year-old man with a history of melanoma presented with a 5.0-cm × 5.5-cm patch with variegated pigmentation (Fig 1, *A*) involving the right zygomatic area. An 8-mm scar from a previous biopsy was present near the lateral aspect of the lower portion of the right eyelid. On dermatoscopy, perifollicular hyperpigmentation with globules was noted adjacent to the scar, near the lateral border (Fig 1, *B*).

## Confocal microscopy appearance

**Fig 2:**
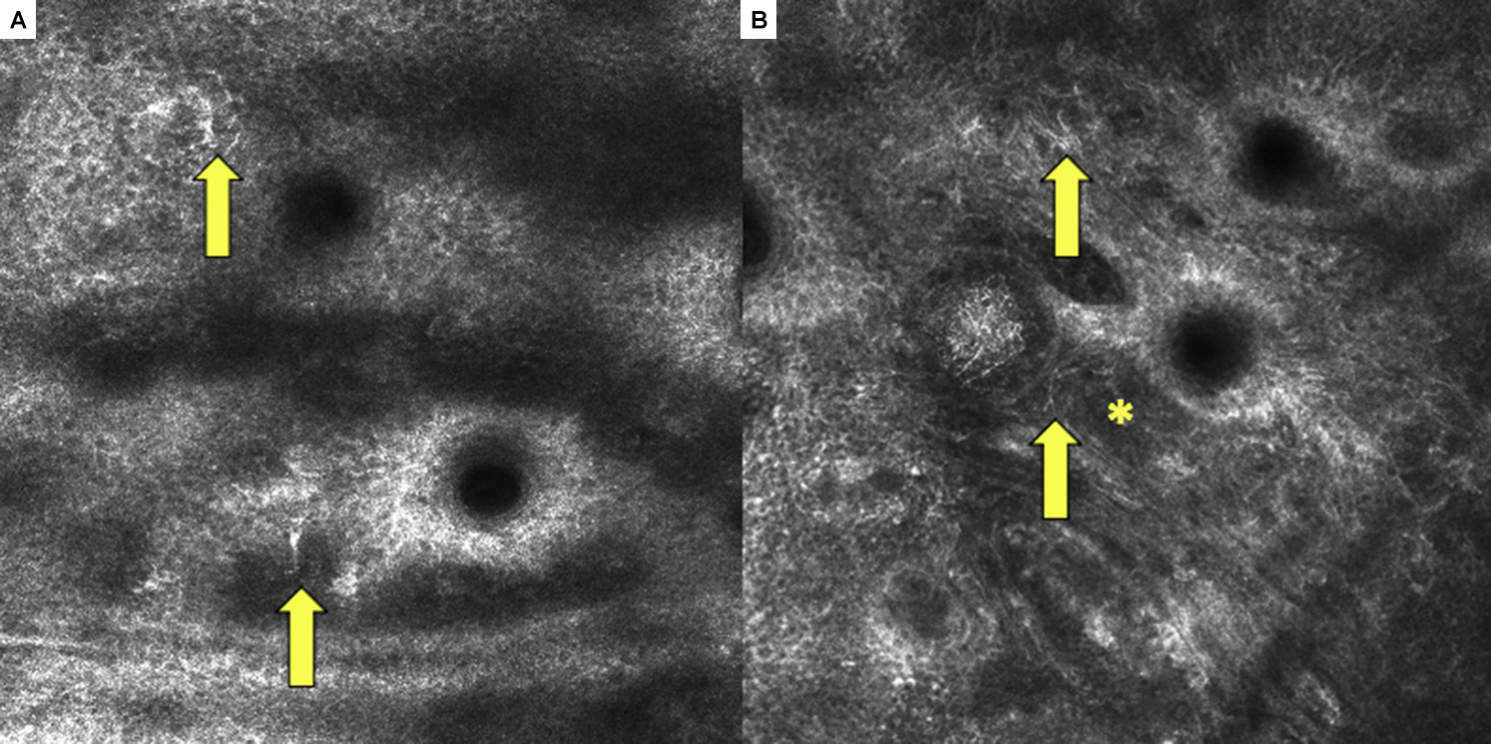
**A**, Reflectance confocal microscopy image (0.75 mm × 0.75 mm, Vivascope 3000; Caliber Imaging and Diagnostics, Inc); location: epidermis. Presence of a predominantly honeycomb pattern and atypical dendritic-shaped pagetoid cells aggregating near follicles (*arrows*), typical for lentigo maligna. **B,** The dermoepidermal junction, showing widespread disarray with the presence of dendritic cells (*arrows*) adjacent to follicles in the background of an unspecified pattern (*asterisk*).

The lesion was evaluated with handheld (Vivascope 3000; Caliber Imaging and Diagnostics, Inc) and traditional reflectance confocal microscopy (RCM) (Vivascope 1500; Caliber Imaging and Diagnostics, Inc). The epidermis demonstrated an irregular honeycombed pattern with the presence of atypical pagetoid cells (Fig 2, *A*). The dermoepidermal junction revealed irregularity with folliculotropism (Fig 2, *B*), suggestive of early lentigo maligna.

## Biopsy technique

**Fig 3:**
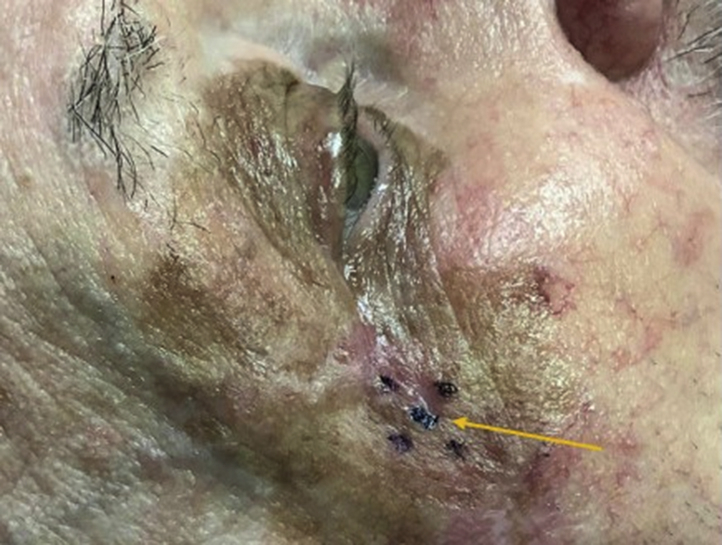
Area of concern identified with handheld RCM stained with a small drop of surgical dye (*arrow*) to serve as a landmark for pathologists. Surrounding marks were made using a sterile marker to delineate the exterior position of the confocal probe’s plastic ending, with central area ink stain correlated with the direct central position of cellular atypia on RCM. *RCM*, Reflectance confocal microscopy.

Assessment with RCM was conducted until concerning features were identified. With the traditional RCM, a dermatoscopic image taken with the attached scope (VivaCam; Caliber Imaging and Diagnostics, Inc) was used to precisely locate the corresponding area of the lesion with suspicious RCM features. Due to the small diameter of the probe for handheld RCM, the suspicious area can be isolated to a 0.75-mm × 0.75-mm area, which can be marked with a marker to correspond with the external border of handheld probe. Once identified, a drop of sterile dye (MarginMarker Ink; VectorSurgical) was applied centrally to serve as a landmark (Fig 3). After 15 seconds, a safe fixative spray was used, followed by deep shave removal.

## Histopathologic diagnosis

**Fig 4:**
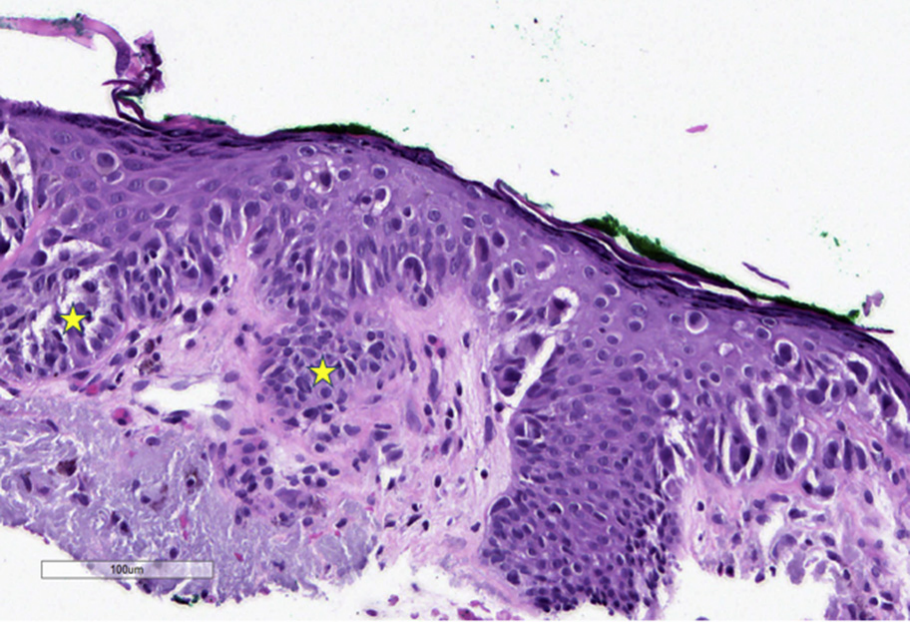
Histologic image showing junctional proliferations of enlarged, atypical melanocytes with hyperchromatic and pleomorphic nuclei arranged in heterogenous nests (*stars*). Green surgical dye used for landmarking noted along superficial epidermis.

The initial biopsy showed scattered melanocytes without confluence or pagetoid spread, and a solar lentigo was diagnosed. Due to high clinical suspicion, an RCM-guided biopsy was taken adjacent to the scar and revealed an asymmetrical proliferation of atypical melanocytes arranged in heterogenous nests (Fig 4). The melanocytes were enlarged, with hyperchromatic to pleomorphic nuclei, and a diagnosis of melanoma *in situ* was made. Green ink was noted by the pathologists.

## Discussion

When evaluating a pigmented lesion suspicious for melanoma, guidelines recommend excisional biopsies; however, partial biopsies may be indicated in large lesions, like those seen in lentigo maligna.^1^ Studies have reported higher rates of sampling error and misdiagnosis with partial biopsies, accounting for over 50% of false-negative melanoma misdiagnoses.^2^ To tackle this issue, Robinson et al^3^ adopted a similar adhesive tape technique previously described for margin mapping large lesions guided by RCM^4^ to navigate a punch biopsy performed in a case of recurrent lentigo maligna; however, this method has several limitations, such as its applicability with handheld RCM and punch biopsies only, restricting its clinical utility. Furthermore, this method relies on the constant repositioning of the tape, which may be time consuming, negating the benefits of rapid handheld RCM evaluation.

Our novel ink-stained RCM-guided biopsy technique is an alternative that is time sensitive, versatile, and simple. This method is applicable with traditional or handheld RCM and may be used to direct excisional or partial biopsies, allowing more widespread utility. RCM evaluation can be conducted like normal, and the application of the dye is quick and simple and does not pose a significant time constraint compared to previously described techniques. Surgical ink used adjunctively with RCM for tumor margin mapping has been described to improve radical excision^5^; however, to our knowledge, we are the first to incorporate rapid ink staining to guide a biopsy.

## Conflicts of interest

None disclosed.

## References

[1] Sladden M.J., Nieweg O.E., Howle J., Coventry B.J., Thompson J.F. (2018). Updated evidence-based clinical practice guidelines for the diagnosis and management of melanoma: definitive excision margins for primary cutaneous melanoma. Med J Aust.

[2] Shao E., Blake T., Po-Chao F. (2020). Prospective study of pigmented lesions managed by shave excision with no deep margin transection of melanomas. Australas J Dermatol.

[3] Robinson M., Soyer H.P., Salkeld A. (2020). A case of recurrent lentigo maligna diagnosed with precise reflectance confocal microscopy-guided biopsy technique. JAAD Case Rep.

[4] Navarrete-Dechent C., Cordova M., Aleissa S. (2020). Use of paper tape to guide reflectance confocal microscopy navigation of large skin lesions. J Am Acad Dermatol.

[5] Venturini M., Gualdi G., Zanca A., Lorenzi L., Pellacani G., Calzavara-Pinton P.G. (2016). A new approach for presurgical margin assessment by reflectance confocal microscopy of basal cell carcinoma. Br J Dermatol.

